# A preliminary study for the assessment of PD-L1 and PD-L2 on circulating tumor cells by microfluidic-based chipcytometry

**DOI:** 10.4155/fsoa-2017-0079

**Published:** 2017-09-04

**Authors:** Jinkai Teo, Anja Mirenska, Meihui Tan, Yifang Lee, Janice Oh, Lewis Z Hong, Richard Wnek, Yoon-Sim Yap, Shian-Jiun Shih, Ali Asgar S Bhagat, Chih-Liang Chin, David AG Skibinski

**Affiliations:** 1 Translational Biomarkers, Translational Medicine Research Centre, Merck Research Laboratories, MSD, Singapore; 2 Clinical Biomarkers, Zellkraftwerk, Bosestrasse 4, D-04109 Leipzig, Germany; 3 Clearbridge BioMedics Pte Ltd, 81 Science Park Drive, The Chadwick, #02–03, Singapore Science Park 1, 118257, Singapore; 4 Translational Molecular Biomarkers, Merck Research Laboratories, Merck & Co. Inc., Rahway, NJ 07065, USA; 5 Department of Medical Oncology, National Cancer Centre Singapore, Singapore

**Keywords:** chipcytometry, circulating tumor cell, CTC, PD-L1, PD-L2

## Abstract

**Aim:**

Expression of PD-L1 in the tumor is associated with more favorable responses to anti-PD-1 therapy in multiple cancers. However, obtaining tumor biopsies for PD-L1 interrogation is an invasive procedure and challenging to assess repeatedly as the disease progresses.

**Materials & methods:**

Here we assess an alternative, minimally invasive approach to analyze blood samples for circulating tumor cells (CTCs) that have broken away from the tumor and entered the periphery. Our approach uses sized-based microfluidic CTC enrichment and subsequent characterization with microfluidic-based cytometry (chipcytometry).

**Conclusion:**

We demonstrate tumor-cell detection and characterization for PD-L1, and other markers, in both spiked and patient samples. This preliminary communication is the first report using chipcytometry for the characterization of CTCs.

## Lay abstract

The proteins PD-L1 and PD-L2 are expressed on some tumors and can inhibit the immune system from attacking and destroying the tumor. Consequently, these proteins are biomarkers for the effectiveness of therapeutic treatments that target this pathway. Here we describe and present preliminary data for a new assay workflow to detect the presence of these proteins on the surface of tumor cells that have broken away from the tumor and entered the blood. Future studies, to develop and validate this assay, would provide a less invasive way of routinely measuring this biomarker than the current practice of taking tumor biopsies.

## Graphical abstract

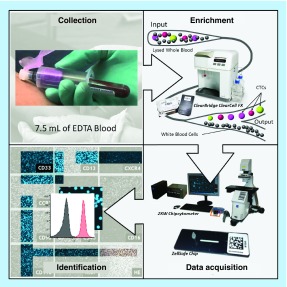

Recent advances in cancer therapy have demonstrated the potential of the immune system in cancer control and rejection. Prominent among these approaches has been the success of anti-PD-1 immunotherapy, which breaks the strong inhibitory signal transmitted by tumor-specific ligands such as PD-L1 to the PD-1 receptor expressed on T cells [1–5]. However, response rates vary widely and thus there is an ongoing effort to identify biomarkers to discriminate among patients who are most likely to gain benefit from these therapies. Identifying nonresponders would also spare them the associated treatment-related toxicities, termed immune-related adverse events, and which include dermatologic toxicity, diarrhea/colitis, hepatotoxicity, endocrinopathies and pneumonitis [6]. A biomarker that has been studied extensively since the first reports of the effects of PD-1 blockade is PD-L1 expression on the tumor. In a number of tumor types, including metastatic melanoma, bladder cancer, non-small-cell lung cancer (NSCLC) and gastric cancer, there is a consistent positive association between PD-L1 expression and an improved response rate [7]. Intratumoral PD-L2 expression has also been shown to correlate positively with response [8]. These findings culminated in the approval of anti-PD-1 therapy to treat patients with NSCLC whose tumors express PD-L1 [9]; nonetheless, a proportion of PD-L1-negative patients also benefit from anti-PD-1 therapy [7]. Given the reported tumor heterogeneity of PD-L1 expression, this observation could be reconciled with the fact that the single core biopsy used to establish patient PD-L1 expression status may not be representative of the whole tumor [10,11]. Furthermore, PD-L1 is not a static biomarker, and the invasive biopsy procedure is not suited to studying how its expression changes during treatment.

Another approach is to detect and characterize PD-L1 expression on circulating tumor cells (CTCs) in peripheral blood samples. CTCs are cancer cells that have broken away from a tumor and entered into the blood circulation and appear to be a strong prognostic factor for overall survival in patients with metastatic breast, colorectal or prostate cancer [12–14]. As CTCs can originate from primary and metastatic tumors, they are believed to give a better representation of the whole disease burden. The expression of PD-L1 on CTCs has been demonstrated in breast cancer patients [15] and lung cancer patients [16]. Interestingly, a recent study found that PD-L1 expression in the nucleus of CTCs was associated with shorter survival durations in colorectal and prostate cancer patients [17]. Furthermore, blood samples, unlike tumor biopsies, can be obtained much less invasively and frequently to facilitate longitudinal monitoring [18]. It has been hypothesized that PD-L1 expression on CTCs may mediate immune escape [19] and a study in NSCLC patients receiving anti-PD-L1 therapy revealed a trend toward a reduction in PD-L1-positive CTCs in patients obtaining clinical benefit [20].

Here we demonstrate a novel workflow for the isolation and characterization of PD-1 ligands, PD-L1 and PD-L2 on CTCs. First, we isolate CTCs from blood samples using the Clearbridge Biomedics ClearCell^®^ FX system, a label-free size-based method that enriches CTCs based on size using inertial and Dean drag forces in a spiral microfluidic device [21–24]. This approach enables rapid and continuous isolation of viable CTCs with reported recovery rates for spiked tumor cells of >85 and 99.99% depletion of white blood cells [21]. Next, chipcytometry, a sensitive single-cell analysis method [25], was used to measure the expression of canonical CTC markers (CD45, cytokeratin (CK), epithelial cell adhesion molecule [EpCAM]), the mesenchymal marker vimentin and PD-1 ligands (PD-L1 and PD-L2) on CTCs. In addition, the incorporation of the fluorescent dye 4′,6-diamidino-2-phenylindole (DAPI) was used to identify nucleated cells. Chipcytometry is a microfluidic chip-based cytometry approach where cells are immobilized on the surface of the microfluidic chip and subsequently stained with fluorescently labelled monoclonal antibodies through iterative cycles of bleaching, staining and imaging [25]. Comparative studies with flow cytometry have established a high degree of correlation between the two technologies [25]. We assessed the feasibility of this combined approach by measuring and characterizing PD-L1 and PD-L2 expression on tumor cells spiked into blood from healthy donors. In addition, we applied the workflow to blood samples collected from breast cancer patients and evaluated the ability to detect and characterize CTCs in these samples. Finally, we discuss the merits and limitation of each technology and the improvement required for developing this workflow into an assay suitable for routine testing in clinical studies.

## Materials & methods

### Cell culture

The lung cancer cell lines H1975 (ATCC^®^ CRL5908™) and A549 (ATCC^®^ CCL185™) were purchased from the American Type Culture Collection (VA, USA). H1975 cells were cultured in Roswell Park Memorial Institute (RPMI) 1640 (Gibco) containing 10% fetal bovine serum (FBS; Hyclone) and penicillin-streptomycin (Gibco). A549 cells were cultured in Dulbecco's modified Eagle medium (Invitrogen, CA, USA) with 10% FBS (Hyclone, UT, USA), 25 mM Hepes (Gibco, MD, USA) and penicillin-streptomycin (Gibco, MD, USA). Cells were cultured at 37°C, 5% CO_2_ and 90% RH.

For stimulation of A549 cells with IFN-γ, A549 cells were cultured in Dulbecco's modified Eagle medium media as described above, seeded into a 12-well tissue culture plate (0.2 million cells/well) and incubated overnight. The following day, the culture media was replaced with fresh media containing a reduced concentration of FBS (2%) and incubated overnight. For the induction of PD-L1 and PD-L2, cells were stimulated with 10 μg/ml IFN-γ (R&D Systems, MN, USA) for 48 h. Cells were harvested with 0.25% trypsin/EDTA for 5 min at 37°C, resuspended in culture media and washed twice with phosphate-buffered saline (Gibco).

### Patients & healthy control samples

The clinical sample collection protocols were reviewed and approved by the Domain Specific Review Boards of the Singapore Health Services (SingHealth, CIRB ref 2014/119/B). Informed and written consent was obtained from all donors prior to blood draw. Three healthy donors and two breast cancer patients’ whole blood samples were collected and stored in 10 ml K2-EDTA tubes (Becton Dickinson [BD], NJ, USA). Clinicopathologic information was recorded for both the patients.

### Flow cytometry

Cells analyzed by flow cytometry (0.3 million cells/sample) were fixed in 200 μl Zellkraftwerk Fixation Buffer and incubated for 15 min at 2–8°C. Fixed cells were centrifuged and washed in 200 μl BD stain buffer (BD). Cells were then centrifuged again and the pellet stained with antibody diluted in 100 μl stain buffer for 15 min at room temperature. All centrifuge steps were performed at 300 rcf for 5 min. Antibodies tested were labelled with phycoerythrin (PE): CD274 (clone 29E.2A3, Biolegend, CA, USA, 1:20 dilution), CD273 (clone MIH18, Biolegend, CA, USA, 1:20 dilution), IgG1 (clone ICIG1, Abcam, UK, 1:10 dilution) and IgG2b, κ (clone MPC-11, Biolegend, CA, USA, 1:20 dilution). Flow cytometry was performed on an FACS Canto system (BD) and 10,000 cells acquired for each sample. flow cytometry standard (FCS) data files were analyzed in FlowJo version 10.0.7.

### Enrichment of CTCs

**Figure 1.**
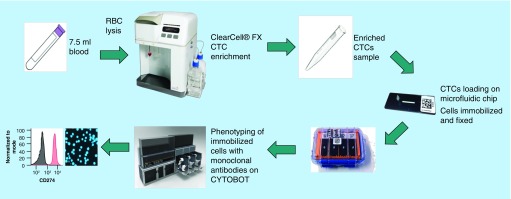
**Novel workflow for circulating tumor cell detection and characterization.** Human whole blood is hemolyzed and centrifuged to harvest nucleated cells. ClearCell^®^ FX enriches CTCs based on size. Enrichment product is loaded onto microfluidic chips and stored at 4°C until ready for analysis. CTCs immobilized on microfluidic chips are phenotyped with monoclonal antibodies against tumor markers using the Zellkraftwerk CYTOBOT. CTC: Circulating tumor cell.

The novel workflow used for the detection and characterization of CTCs is shown in Figure 1. Whole blood was collected from breast cancer patients or healthy controls in 10-ml K2-EDTA tubes (BD). For spiked experiments 7.5 ml healthy control blood was spiked with approximately 200 H1975 cells (exact number was determined by counting under a microscope prior to spiking). A red blood cell (RBC) lysis step was performed using RBC Lysis Buffer (G Biosciences, MO, USA). After RBC depletion, the cells were pelleted by centrifugation and resuspended in 4.3 ml of resuspension buffer (Clearbridge BioMedics, Singapore). The resuspended cells were loaded onto the ClearCell FX system for automated CTC enrichment. The enrichment product was harvested by centrifugation (300 × *g*, 4°C) and resuspended in Zellkraftwerk wash buffer.

### Chipcytometry

Prior to sample loading, Zellsafe™ chips (Cat. number 28050606/01–010) were rinsed three-times each with 100 μl Zellkraftwerk wash buffer. Once loaded onto the chip, cells were fixed by rinsing five-times with 100 μl Zellkraftwerk Fixation Buffer and incubated for 15 min at 2–8°C. Loaded chips were rinsed five-times with 100 μl Zellkraftwerk Storage Buffer for long-term storage at 2–8°C. Chipcytometry was performed as described previously [25] with each antibody/DAPI stained separately through iterative cycles of bleaching, staining and imaging. Staining for all antibodies was at a volume of 300 μl diluted in phosphate-buffered saline and incubated 15 min at room temperature. For the staining with the antipan-CK-antibody, the antibody was diluted in 1 × FOXP3 Perm Buffer (Biolegend) and cells were rinsed with 1 ml of this buffer prior to staining. For DAPI staining, cells were incubated with FOXP3 Fix/Perm Buffer (Biolegend) for 20 min and DAPI staining was subsequently performed as previously described for the antipan-CK antibody. Antibodies used were as follows: CD45-PerCP-Cy5.5 (clone HI30, Biolegend, surface staining, dilution 1:500), CD326 (EpCAM)-PE (clone VU-1D9, Thermo Fisher Scientific, MA, USA, surface staining, dilution 1:1000), pan-CK-AF488 (clone C-11, Cell Signaling Technology, MA, USA, intracellular staining, dilution 1:250), CD274-PE (clone 29E.2A3, Biolegend, surface staining, dilution 1:500), CD273-PE (clone MIH18, Biolegend, surface staining, dilution 1:100), Vimentin-PE (clone D21H3, Cell Signaling Technology, MA, USA, intracellular staining, dilution 1:500). To identify the appropriate antibody concentrations, A549 cells (either unstimulated or stimulated with IFN-γ) were used. Image and data processing was performed using Zellkraftwerk's chipcytometry data pipeline and exported to FCS format for further analysis using FlowJo v10.0.7 (Tree Star, OR, USA). Fluorescence is background subtracted from the unstained image and normalized with respect to the cell size.

## Results

### Establishment of PD-L1 & PD-L2 analysis by chipcytometry

**Figure 2.**
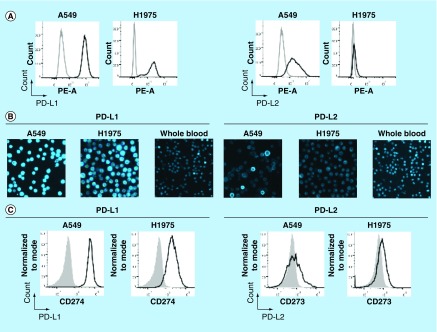
**Establishment of PD-L1 and PD-L2 analysis by chipcytometry.** **(A)** Flow cytometry analysis. Non-small-cell lung cancer cell lines A549 and H1975 were incubated with antihuman PD-L1 and PD-L2 (black), and corresponding isotype control (gray). Cells were fixed prior to staining. **(B & C)** Chipcytometry analysis. A549 cells (black), H1975 cells (black) and whole blood samples collected from a healthy donor (gray) were loaded onto Zellkraftwerk ZellSafe™ chips, fixed and stained with antihuman PD-L1 and PD-L2.

Flow cytometry was used to establish the specificity of the selected monoclonal antibodies for PD-L1 and PD-L2 (clones 29E.2A.3 and MIH18, respectively) on the NSCLC cell lines A549 and H1975 (Figure 2A). To upregulate PD-L1 and PD-L2, A549 cells were incubated with IFN-γ for 48 h prior to staining [26]. Flow cytometry analysis demonstrated the presence of PD-L1 on A549 and H1975 cells, and PD-L2 on A549 cells (Figure 2A). These observations are in line with previously reported observations of PD-L1 and PD-L2 expression on these cell lines [26,27]. The specificity of the staining was demonstrated by the fact that no signal was detected for the isotype control on both A549 and H1975 cells. PD-L2 was weakly expressed on H1975 cells.

Next, we established the detection of PD-L1 and PD-L2 on A549, H1975 and whole blood by chipcytometry, a microfluidic-based cytometry approach where cells are immobilized on a microfluidic chip. Immobilized cells were stained with fluorescently labelled antibodies and a fluorescent light image acquired (Figure 2B). Median fluorescence intensity per cell was calculated and depicted as a histogram (Figure 2C). Whole blood was used as a biological comparison control to distinguish expression on A549 and H1975 cells to background levels [28]. The relative expression of PD-L1 and PD-L2 detected on H1975 cells by chipcytometry was consistent with that observed by flow cytometry (Figure 2A & C). For A549 cells, while detection of PD-L1 by chipcytometry was consistent with that observed by flow cytometry, detection of PD-L2 was reduced. A549 cells expressing PD-L2 were still detectable by chipcytometry.

### Detection of spiked tumor cells

**Figure 3.**
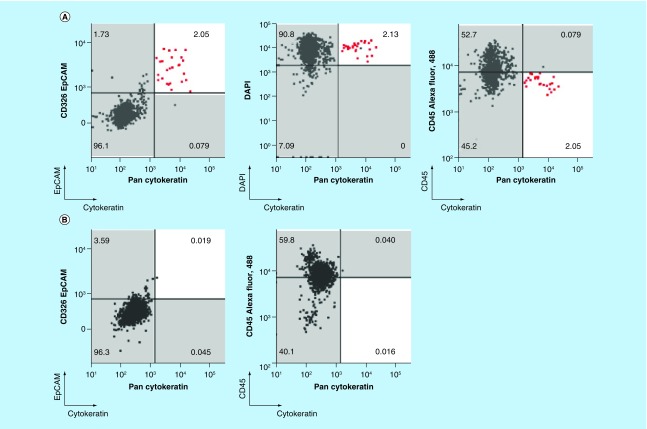

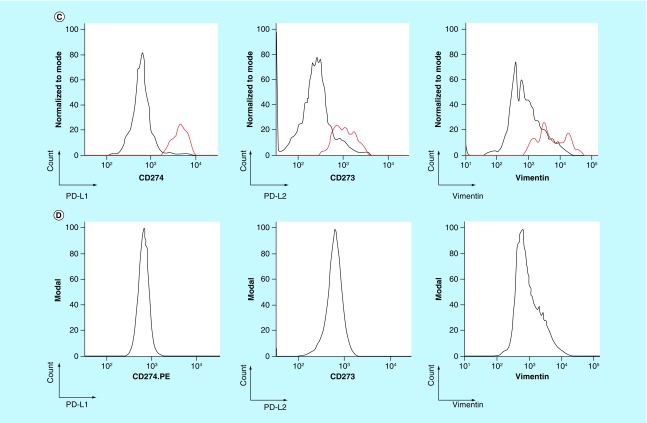
**Microfluidic chipcytometric detection and characterization of spiked tumor cells.** CK, EpCAM, nuclear, CD45, PD-L1, PD-L2 and vimentin staining were used to distinguish tumor cells (population highlighted in red) from the white blood cell population after enrichment on the ClearCell FX^®^ system. **(A & C)** Whole blood spiked with H1975 tumor cells. **(B & D)** Whole blood not spiked with H1975 tumor cells. **(A & B)** H1975 cells gated as CK^+^, EpCAM^+^, DAPI^+^ and CD45^-^. **(C & D)** Histograms of PD-L1, PD-L2 and vimentin expression in H1975 tumor cells (red) and white blood cell contaminants (black). CK: Cytokeratin; EpCAM: Epithelial cell adhesion molecule.

**Figure 4.**
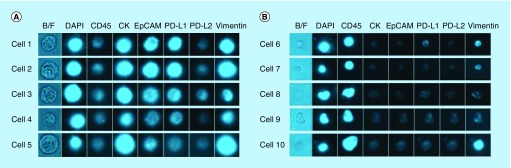
**Representative images from the microfluidic chipcytometric detection and characterization of spiked tumor cells.** **(A)** H1975 cells detected in the spiked samples. The staining profile allows us to distinguish these cells from the white blood cells. **(B)** Representative images of white blood cell contaminants present after enrichment by ClearCell FX^®^ system.

To evaluate the capability of chipcytometry to detect rare tumor cells isolated using the ClearCell FX system, we performed spiking experiments by adding approximately 200 H1975 cells to 7.5 ml of whole blood. Spiked blood samples were enriched for tumor cells using the ClearCell FX system and subsequently loaded onto microfluidic chips for analysis by chipcytometry (see Materials & methods). Identification of tumor cells in these spiked samples was achieved by staining the isolated cells for DAPI, CK and EpCAM. Although, as expected with the ClearCell FX system, CD45^+^ cells were present in the enrichment product, DAPI^+^ CK^+^ and EpCAM^+^ cells were clearly distinguishable from these white blood cell contaminants (Figure 3A). As a control, these cells are not observed in distributions for blood not spiked with tumor cells (Figure 3B). In addition, through subsequent iterative cycles of bleaching, staining and imaging we were able to demonstrate the feasibility to detect PD-L1, PD-L2 and Vimentin on the spiked H1975 cells (Figure 3C). Cells expressing PD-L1, PD-L2 and Vimentin were not observed in distributions for blood not spiked with tumor cells (Figure 3D). Mean recovery of the tumor cells in the spiked samples was 22.8% ± 5.4% (Table 1). Fluorescence microscopy images of each cell acquired during the chipcytometry analysis highlight and confirm the detection and phenotype of the tumor cells (Figure 4A & B).

**Table 1.** **Recovery of H1975 cells as identified by Zellkraftwerk chipcytometry after enrichment of spiked blood samples by ClearCell^®^ FX system.**

**Donor**	**Spike amount^†^**	**Chip average^‡^**	**Percentage recovery by donor (± 95% CI)^§^**	**Total percentage recovery (± 95% CI)^¶^**
1	213	29.5	27.7 ± 10.0	22.8 ± 5.4

2	186	19	20.4 ± 9.2	

3	191	19.5	20.4 ± 9.1	

^†^Blood was spiked with a known amount of H1975 cells and enriched for CTCs via the ClearCell FX system. After enrichment, the cells from each donor were split and each half loaded into a separate Zellkraftwerk ZellSafe™ chip according to standard Zellkraftwerk's protocol and stored at 4°C until analysis.

^‡^Standard error of chip average was calculated by assuming an underlying Poisson distribution.

^§^The 95% CI was calculated by using a normal distribution to approximate the Poisson.

^¶^The total recovery is calculated from the mean and variance of each donor, under a Poisson distribution and assumption of independence. The 95% CI was calculated by approximating a normal distribution.

CTC: Circulating tumor cell.

### Detection of CTCs in cancer patient samples

**Figure 5.**
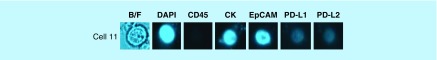
**Example images of a circulating tumor cell isolated from a breast cancer patient.** Blood from the patient was processed for CTC enrichment using the ClearCell^®^ FX system. The sample was then analyzed by chipcytometry and a single CTC determined to be PD-L1/PD-L2 negative was detected. CTC: Circulating tumor cell.

With the aim of evaluating the feasibility of detecting CTCs in ‘real world’ patient samples, peripheral whole blood samples were collected from breast cancer patients (Table 2) and subsequently processed for CTC enrichment using the ClearCell FX system. The samples were then analyzed for the presence of CTCs and PD-L1/PD-L2 expression by chipcytometry. We detected a single CTC in patient A and determined the PD-L1 and PD-L2 status of this CTC to be negative (Figure 5).

**Table 2.** **Clinicopathological characteristics of metastatic breast cancer patients who provided samples for this study.**

**Patient sample number**	**Age**	**Gender**	**Cancer stage**	**Treatment history**
A	43	Female	4	Docetaxel and cyclophosphamide (AC) chemotherapy, herceptin

B	58	Female	3	Surgery

AC: Adriamycin cyclophosphamide.

## Discussion

We have assessed a novel assay workflow to detect CTCs and characterize PD-L1 and PD-L2 expression on these cells in peripheral blood samples. Specifically, in this study, we established chipcytometry as a method for the detection of PD-L1 and PD-L2 on tumor cells using cancer cell lines A549 and H1975. Spiking experiments revealed that our workflow could detect tumor cells in whole blood samples with a mean detection rate of 22.8% (± 5.4%). In addition, we could determine their PD-L1 and PD-L2 expression levels. CTC detection and PD-L1/PD-L2 assessment was also demonstrated on blood samples from patients with breast cancer. We believe that further development and clinical validation of this assay would allow us to monitor the dynamics of PD-L1 and PD-L2 expression during anti-PD-1 therapy and explore whether expression of these biomarkers is associated with response. It would also be of interest to establish, through paired blood and tumor biopsy samples, to what extent expression of PD-L1 and PD-L2 in CTCs reflects what is seen on the tumor biopsy.

The novel workflow described here combines two different technologies that tackle the challenges of isolating and characterizing CTCs. The label-free ClearCell FX system is preferable to antibody-based affinity approaches given the known heterogeneity in biomarker expression of CTCs [29]. The system is well established [21–24] and the simplicity and robustness of the device is well suited for use in a clinical setting. For downstream analysis of the isolated CTCs, we believe the main advantages of chipcytometry lie in the iterative staining process that allows retrospective evaluation of additional markers and the potential to measure a large number of parameters without the spillover/compensation problems encountered with flow cytometry. This approach allows the analysis of additional immunomodulatory targets on tumor cells beyond PD-L1 and PD-L2, which is particularly critical, considering high dimensional analysis of these markers is likely to become increasingly relevant as immunotherapy moves beyond the administration of single immunomodulatory agents toward combinations that synergize in their antitumor immune response. In addition, the possibility of including more tumor and immune markers (positive and negative) will increase confidence that the identified cells are indeed CTCs [16].

In this preliminary communication, we demonstrate a workflow utilizing the CTC enrichment and chipcytometry technologies described. However, these data are preliminary, and although it encourages future development of the assay, further experimentation is required to fully establish feasibility of this approach. Despite the virtues of the technologies utilized, we appreciate that further development and optimization is required to have an assay suitable for a widescale application in clinical studies. For example, given the modest recovery reported in our feasibility study, further studies are needed to understand which steps in the workflow are responsible for the loss in recovery. We speculate that losses likely occur from manipulations downstream of CTC enrichment as the ClearCell FX system has previously reported recovery rates of >85% [21]. These downstream manipulations include sample centrifugation, buffer replacement, chipcytometry loading and chipcytometry washing steps. Other areas of optimization include evaluation of blood collection tube compatibility, with both the enrichment and chipcytometry technologies, and more extensive optimization of antibody clone selection, staining concentration and stain sequence. The need for further experiments to elucidate where these losses in CTC recovery occurred cannot be understated, and will be the subject of future studies. Detecting only a single CTC in one patient and no CTCs in the other patient is lower than that has been reported for breast cancer patients [15], and a number of factors could have contributed to this, including the modest recovery for the spiked samples and low patient sample number in this study. Furthermore, the treatment received by the cancer patients could have affected the numbers of CTCs in circulation, and samples taken from patients prior to treatment may be more appropriate for evaluating the feasibility of new assay workflows. Expression of PD-L1 and PD-L2 in lung cancer tumors is known to be heterogeneous, with some patients expressing a high proportion of tumor cells positive for these markers while others have no detectable PD-L1 and PD-L2 [30]. Finally, exploration of an appropriate analysis strategy and software platform will facilitate identification of CTCs without restricting their identification to the classical definitions used here (DAPI^+^, CD45^-^, CK^+^, EpCAM^+^). This will become especially relevant as we consider the quantitative measurement of each marker's expression and add higher numbers of markers to the assay.

## Conclusion

Here we demonstrate a novel workflow, the first using chipcytometry, to isolate CTCs from patient blood samples and subsequently characterize them for the expression of PD-L1 and other markers. Spiking experiments revealed that our workflow could detect tumor cells in whole blood samples with a mean detection rate of 22.8% (+/- 5.4%). We demonstrate CTC detection and PD-L1 and PD-L2 expression assessment on blood samples from patients with breast cancer.

## Future perspective

The expression of PD-L1 on the tumor has been established as a diagnostic for treatment with anti-PD-1 therapy in lung cancer. Future work will establish the utility of measuring the expression of PD-L1 on CTCs and whether this is reflective of expression in the tumor and predictive of response to treatment. In addition, the dynamics of PD-L1 expression during treatment will be determined and the value of doing this in monitoring patient response understood. Isolation and characterization of CTCs will be explored as a potential avenue for longitudinal tracking of this biomarker where invasive serial biopsies are not possible. Furthermore, as immunotherapy is extended to more targets and combinatorial approaches are shown to improve therapeutic benefit, new technologies and workflows will be established to routinely capture these rare cells and fully characterize them using high dimensional proteomic and genomic approaches.

**Executive summary**

**Background**

- The expression of PD-L1 in the tumor has been associated with more favorable response rates to anti-PD-1 therapy.
- However, obtaining tumor biopsies for PD-L1 interrogation is an invasive procedure and challenging to assess repeatedly as the disease progresses.
- A potential alternative, minimally invasive, approach is the analysis of blood samples for circulating tumor cells (CTCs) that have broken away from the tumor.
- A novel workflow for the detection and characterization of CTCs from whole blood was assessed for feasibility.

**Experimental**

- Blood from healthy donors and cancer patients was collected in 10 ml K2-EDTA tubes. Healthy donor blood was spiked with H1975 cells to evaluate feasibility of the approach.
- The ClearCell^®^ FX System, a sized-based microfluidic enrichment system, was used for enrichment of CTCs.
- CTC detection and characterization for PD-L1 and PD-L2 was performed using microfluidic-based cytometry (chipcytometry).

**Results & discussion**

- Spiking experiments revealed that our workflow could detect tumor cells in whole blood samples with a mean detection rate of 22.8% (+/- 5.4%).
- In a patient with breast cancer, a single CTC was detected and the PD-L1 and PD-L2 status of this CTC determined to be negative.

**Conclusion**

- We demonstrate preliminary assessment of a novel workflow, the first using chipcytometry, to isolate CTCs from patient blood samples and subsequently characterize them for the expression of PD-L1 and other markers.
